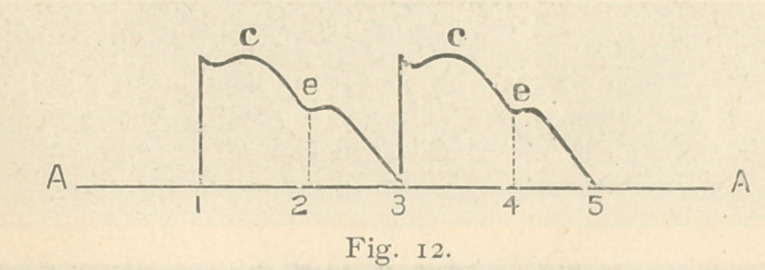# The Pre-Albuminuric Stage of Chronic Bright’s Disease

**Published:** 1885-05

**Authors:** Charles W. Purdy

**Affiliations:** Member of the Chicago Academy of Sciences. First Vice-President of the Chicago Medical Society; 163 State Street


THE CHICAGO

### MEDICAL JOURNAL AND EXAMINER.

VOL. L. MAY, 1885. No. 5.

### ORIGINAL COMMUNICATIONS.

ARTICLE I.

### THE PRE-ALBUMINURIC STAGE OF CHRONIC BRIGHT'S DISEASE.

*By* CHARLES W. PURDY, M. D. *Member of the Chicago Academy of Sciences. First Vice-President of the Chicago Medical Society.*

The more closely one studies the clinical history of chronic Bright's disease, the more apparent it becomes that in its early stages albumen is quite often absent from the urine.

That I may not subsequently be misunderstood by confusion of terms, I will here premise, that what I shall have to say upon this subject is intended to apply only to that group of clinical symptoms which are accompanied or preceded by the granular kidney. Such a condition of the kidney may exist; nay, it may even pass through all the stages of contraction, with its usual clinical symptoms, till death results, without albumen making its appearance in the urine. Such cases are by no means rare, as the records of post-mortem rooms in our hospitals demonstrate, which I shall later on amply verify.

Furthermore, I am convinced that a well-defined pre-albuminuric stage of renal cirrhosis exists, which it is possible to make out, by well-defined and more constant symptoms, than that of the presence of albumen in the urine. This idea is by no means new, but has been long suspected, and is making itself felt with increasing force each year, if we are to judge from the increasing contributions to the literature of the subject, which have appeared within the past fifteen years. *

It has become the dictum since the days of Bright, to hinge the diagnosis of all diseases bearing his name on the one symptom, namely, the presence of albumen in the urine. Now the diagnosis of Bright's disease embraces a much more comprehensive investigation of facts and data than the mere search for albumen, the neglect of which leads daily to very unfortunate results.

Our life insurance companies are imposed upon frequently through incomplete so-called examinations of patients whose risks are passed as "first-class" (and some of them are literally so), upon the almost sole presumption, so far as the kidneys are concerned, that no disease exists because no albumen is present in the urine. Many of these cases are hurrying on to contracted renal organs, uremia, and death, which can at best be postponed but a short time. Nor is this picture an overdrawn one, as the following case illustrates. A year since a man made application to one of our leading insurance companies for insurance on his life. The medical officer representing the company found a slight trace of albumen in his urine, and he was referred to me. Atheromatous vessels, which had a year ago been manifest through an obstinate epistaxis, showed the usual degenerative

* Contributions on this subject, by Drs. Southy, Burdon-Sanderson, Mahomed, Saundby, and others, have appeared.

changes accompanying renal cirrhosis, to have been in progress for some time. Fig. 1 shows his pulse tracing, which is characteristic.

Fig. 1.

He was put under treatment, and properly restricted dietary, and told that his disease was incurable, but with care he might live some months, or perhaps years.

During my absence abroad, this man applied to another company for insurance, and was passed by the medical officer, and his life was insured for $8,000. His kidney disease is now slowly but surely advancing towards renal atrophy; and this is by no means the first case of the kind which has come under my notice.

In the days when I looked upon the presence of albuminous urine as the essential diagnostic symptom of renal disease, I have watched cases closely *for years*, suspecting the stealthy approach of cirrhotic kidneys, but was lulled into contentment by an occasional examination of urine, which was found to be free from albumen, till at length an attack of renal dyspnœa, or some other evidence of the disease, had pointed out that which I had suspected had really been going on under my very eyes. I have just now in mind a case which I had watched and suspected for nearly two years, but never could find a trace of albumen in the urine. Gastro-intestinal disturbances at length appeared, and finally a severe and prolonged attack of recurrent dyspnœa aroused me to the realization that my patient was suffering from renal cirrhosis; and subsequent autopsy proved the correctness of the diagnosis. Now I do not wish to be understood as underestimating the value of albuminuria as a symptom of renal disease; on the contrary, *when present*, I attach the greatest importance to it, under certain conditions. But what I contend for is that when albumen is absent from the urine it does not prove that no renal disease is present. In other words, it is misleading to teach that the diagnosis should hinge upon this symptom alone. The form of renal disease under consideration is the very one in which albumen is most likely to be absent.

It is to be feared that our recent rapid advance in pathological knowledge may have tended to incline medical thought too closely to the structural to the expense of the general disorder. In other words, the seat of disease itself is scanned most minutely and laboriously, perhaps at the expense of less reliance on the good old clinical methods of investigation; for one of our great teachers has said: "After all, pathology is only a study of results, *not causes*, of disease."

It was long ago noted by writers that in certain numbers of cases of renal disease, albumen disappeared from the urine. Indeed, this did not escape the attention of Richard Bright himself, who was the first to note this circumstance. But others, among them Christoson, Rayer, Rees and Malmsten, confirmed the observation.

More recent authors point out that the cirrhotic form is the one in which this condition is noted, and pretty much all of them agree that in the early stages albumen is quite likely to be absent from the urine.

Professor Grainger Stewart, in his admirable work on "Bright's Disease," page 188, says, referring to cirrhosis of the kidney: "The early symptoms are very slight, such indeed as might easily escape notice. I have seen several cases in which the renal lesion was sufficiently distinct on post-mortem examination, but in which there had been no albuminuria and no dropsy during life;" and again, page 189, he writes: "In the early stages (of cirrhosis) albumen is occasionally present, generally in very small quantity. One day it may be distinct, but the next day we search for it in vain."

Dr. Roberts, in his last edition, says: "But it must be admitted that chronic degeneration of the kidneys, not distinguishable from some forms of Bright's disease, does exist under certain circumstances, without albuminuria."

Bartels says (Ziemssen's Cyclopedia, page 431, Vol. XV.): "It is these cases of genuine contracting kidney, that urine is occasionally excreted, which is in no way to be distinguished from that secreted by healthy kidneys;" and again, on the same page, 435–436, he adds: "But albuminuria, as later experiences in this direction have taught me, is no constant symptom in this affection. The temporary absence of albumen, I have witnessed repeatedly,—for purpose of diagnosis, the matter is one which deserves to be detailed at greater length." On page 440 he cites a case which came under his care in the hospital, and continued under treatment, from Jan. 26th till death, March 3rd, and though both kidneys proved to be cirrhotic and extremely contracted at the autopsy, he says: "Albumen was entirely absent from the urine throughout."

Dr. Geo. Johnson, in his "Lectures on Bright's Disease," page 52, says: "The amount of albumen varies considerably. Absent or scanty in the early stage, it may be rather copious in the middle periods, and again scanty, or even entirely absent, in the stage of extreme degeneration of the kidney."

Dr. Dickinson, in his "Treatise on Albuminuria," says, in referring to the urine in the early stage of granular degeneration: "If examined in this early stage, it may be found perfectly free from albumen, or may contain only a minute trace, or, a trace only after food, or on getting up in the morning;" and again he observes: "Early in this disease, the urine is free, both from albumen and casts; later on, a trace of albumen appears, which may not be discoverable until the constitutional symptoms are such as to indicate an advanced stage of the disease. So little albumen may be present, even to the end, that care is needed for its detection. And even that little may not be constant, but discoverable only after sleep, or meals."

Dr. Tyson, in his work on "Bright's Disease and Diabetes," page 178, says, in referring to the urine in cases of interstitial nephritis: "The albumen is small in amount, and may be temporarily absent, or it may be absent before a meal, and present after it."

Dr. Millard, in his treatise on "Bright's Disease," page 141, says: "The fact is, however, that in chronic nephritis, especially the interstitial, the appearance of albumen is often preceded for considerable, and even for a very long time, by morbific changes in the kidney, which are not recognized until the appearance of albumen. Indeed, nephritis may exist to such an extent as to produce even cirrhosis, without albumen *ever* making its appearance in the urine." He also very correctly adds, (page 143): "To rely upon albumen, solely as a means of determining the existence or non-existence of nephritis, is to rely upon an *ignis fatuus*. It is at best a coarse and primitive test of its presence; insufficient in itself, and unsatisfactory in comparison with more searching and absolutely accurate means of diagnosis." Dr. Millard cites several cases in his experience, wherein albumen was entirely absent from the urine, throughout the course of renal cirrhosis; and, indeed, he devotes a special chapter to the consideration of "Nephritis without Albuminuria;" which is well worthy of careful perusal.

In "*Guy's Hospital Reports*," for 1879, (page 367) will be found an analysis of one hundred cases of granular kidneys, observed in that hospital, previous to that year. Out of the hundred cases noted, the very large proportion of seventy-four were characterized by the absence of albumen from the urine. Dr. Saundby, on "Occurrence of Dropsy in Granular Kidney," introduces a table showing the diagnosis—which was sent down from the wards, to the post-mortem room, at the Birmingham General Hospital*—in ninety-eight cases, in all of which, the kidneys proved to be granular. Out of these ninety-eight, only twenty-two were sent down with the diagnosis of Bright's disease. Hence, we may fairly assume, that in seventy-six of them, albumen was not discovered in the urine. Again, Dr. Mahomed collected the records of sixty-one cases treated in Guy's Hospital during the two years 1879 and 1880.† In forty-one of these, albumen was never discovered in the urine. Now, putting these statistics together, we have two hundred and fifty-nine cases of chronic Bright's disease, in which albumen was present in the urine only in sixty-eight cases, leaving one hundred and ninety-one cases, or about seventy-four per cent., in which albumen was absent. These cases can be open to little question, for, be it remembered, they were all hospital cases, subject to the usual rules for daily examinations of urine, and the diagnoses *in all* were confirmed by autopsy.

In the light of such unanswerable testimony as the foregoing, I maintain that the mere absence of albumen from the urine, constitutes no reliable evidence of the non-existence of chronic Bright's disease, and above all in its early stages. If clinical evidence, conjoined with post-mortem examination of our cases, teaches us anything, it is, that this disease may exist, and progress through years, without giving rise to albuminous

* Birmingham Medical Review, April, 1881.

† Guy's Hospital Reports, Vol. XXV, 1880 and 1881, pages 304 and 305.

urine; and therefore, a clinical symptom so notoriously inconstant and unreliable, even in the fully formed stages of the disease, constitutes the most flimsy evidence upon which to construct the diagnosis of a disease, the most insidious and silent in its early advances of any with which medical science has to deal.

In many instances chronic Bright's disease is a condition in which its early steps have been so slow and imperceptible that half the lifetime of the individual has been consumed in leading up to it through continuous functional overtaxing of the gland before any marked textural changes can be made out; yet, in this early stage, other evidences of the disease are to be found, which, taken collectively, will establish the diagnosis in the majority of cases.

I do not mean to say that absolutely the first departure from the normal condition is to be made out, for from the very nature of the first changes in the gland, it is probable that these escape detection. But I hold that during that stage in which no appreciable change can be made out in the renal epithelium (which may extend over years), albumen, as a rule, is absent from the urine, and yet, through other symptoms, the presence of the disease may often be revealed.

I have examined, microscopically, many sections of cirrhotic kidneys, including those in my own collection and in the Pathological Laboratory at Aberdeen, as well as in other places, and in the early stages of this disease I find little, if any, departure from the normal in the tubular epithelium. The earliest changes which I am able to make out, seem to be slight thickening of Bowman's capsule, sparse septa of apparently new connective tissue creeping in at varying points from the capsule and between the interlobular arteries, as well as increased thickness of the coats of the small arterial vessels. I

have compared, side by side, sections of early cirrhotic kidneys with those of normal, with the view of ascertaining the true condition of the epithelium lining the convoluted tubes in the former, and I cannot see that it varies essentially from the normal. It is misleading to speak of cloudiness of the renal epithelium. That is the normal condition, it is never clear unless cleared by some chemical agent. The very first change I am able to make out is the presence of some fat globules in the epithelial cells, and perhaps a slight flattening of the latter, so that the lumen of the tube is somewhat more open. Desquamation of the epithelium, if it ever occurs in this disease, certainly does not do so in this early stage.

Bartels says: "This much, however, I believe to be beyond all doubt, namely, that genuine renal contraction—the so-called third stage of Bright's disease of our (German) writers, Grainger Stewart's cirrhosis of the kidney, is the result of a primary growth or proliferation of the intertubular connective tissue." Dr. Dickinson, in his treatise on "Albuminuria," says: "I have examined these cells (tubular epithelial) in a great number of granular kidneys, and have carefully drawn their outlines and dimensions, as seen through an one-eighth inch object-glass. The conclusion I long ago formed, one which has been justified by careful and continued observation, is this: In the vast majority of cases, in all cases excepting those in which the contraction of the organ has become extreme, *the epithelium is exactly such as is found in healthy kidneys.*"

Charcot, in his "Lectures on Bright's Disease of the Kidneys," describes minutely the changes found in what he terms the "first stages of interstitial nephritis," as follows: "From the commencement the connective stroma is infiltrated with a more or less considerable quantity of small cellular elements, which, following the theory adopted, are called leucocytes or embryonic cells. To this cell infiltration is due the grayish yellow color sometimes presented by the kidney at this stage of the disease. Another characteristic revealed by histological study is, that at this period the tubular apparatus of the kidney presents no appreciable alteration ; the epithelium in the convoluted tubes *is in its place, and perfectly healthy.*"

This, then, explains the non-appearance of albumen in the urine during the early stage of granular kidney, for pathological and clinical evidence shows that those diseases which cause destruction of the renal epithelium are the ones which give rise to the most constant and profuse amount of albuminuria.

On this point Senator says : "The normal functions of epithelial cells is assigned as the reason for the absence of albumen in the secretion of the liver, the most prominent of the second class of glands, and likewise in the secretions of the perspiratory and lachrymal glands, supposing it to be the case that these latter secretions in their pure state are really non-albuminous. If the urine be regarded simply as a true non-albuminous glandular secretion, the epithelium must be credited with the function of preventing the escape of albumen from the blood. Even if the urine be regarded as a mixture of a transudation with a glandular secretion, the latter at least being the produce of the epithelium of the uriniferous tubes, must for the same reason be considered to be non-albuminous ; whatever view may be entertained as to the presence or absence of albumen in the transudation from the glomerular vessels. The conclusion is forced upon us, that when their nutrition and functions are disturbed, or when the epithelial cells of the uriniferous tubes are in a state of complete decay, albumen will escape from the blood and lymph, and show itself in the urine hitherto apparently non-albuminous ; that is, that albuminuria will become developed. Observations which are alleged to prove the contrary must be based upon error or defective investigation, for if not, all our doctrines with regard to specific glandular secretion must be thrown to the winds."

It may be asked, then, if the renal epithelium is not the seat of any degenerative change in renal cirrhosis? How does it happen that albumen *is* present in the urine *sometimes* in the course of the disease?

We answer, that the latter is due to increased intra-vascular pressure, which is brought about as a legitimate outgrowth of the disease.

It has been established, beyond doubt, that increased blood pressure within the vessels, will, under certain conditions, cause albumen to appear in the urine. J. B. Stockvis showed by extended experimental and clinical investigations, that alterations of the circulation which checks the afflux of arterial, or the escape of venous blood, causes albumen to pass into the urine. In other words, over-fullness of the venous circulation, or slowing of the current, produces albuminuria Since, however, other observers, and especially Senator, has pointed out that high pressure in the arterial vessels will also cause albuminuria, though to a less extent.

Active muscular exertion is accompanied by an increase of the muscular power of the heart's action by about twenty-five per cent., hence, the intra-vascular pressure is increased to that extent in the arteries, and it is no uncommon thing to find albumen in the urine of healthy men, who are undergoing active muscular exercise. Heat also causes a direct rise in the arterial blood pressure, as shown by the dilated, and turgescent vessels everywhere; as a consequence of this, we often note appearance of albumen in the urine during febrile conditions. Senator proved the proposition by the following experiments. He introduced rabbits into an oven, constructed for the purpose, previously having drawn off their urine, and testing it for albumen. He then slowly increased the heat in the chamber, and again tested the urine, and he states as to these experiments, many times repeated, that, "The result in all cases was the production of albuminuria, when the bodily temperature had been increased by 1.5°–3°C., with sufficient rapidity, or the heat continued for a sufficient length of time."

It may be observed that heat, or any other agent, *suddenly applied*, which raises the arterial pressure, causes a contraction of the capillaries which resists the increased pressure, and albumen does not transude under such circumstances. The pressure must be gradual to cause albuminuria. We have many examples of the production of albuminuria by intravenous pressure. Bartels relates a case bearing on this point. It was one in which there was impeded escape of the blood of the renal vein, in consequence of thrombosis of the inferior vena cava, occurring in a very robust man, forty-four years of age. Bartels says: "This patient passed *enormous quantities* of urine, and with a specific gravity varying from 1.011 to 1.013, and always containing albumen!" The occurrence of albumen in the urine as a consequence of valvular lesions of the heart, is another well known example of this kind, leading to what has been called cyanotic induration of the kidney; a condition, by the way, very similar to renal cirrhosis.

Thus it may be laid down as a rule, that intra-vascular pressure, whether arterial or venous, if only of sufficient extent, is likely to result in albuminuria.

Now, in the advanced stage of granular kidney, we have at least two conditions which lead up to increased blood pressure, and especially so within the glomerular vessels. First, hypertrophy of the left ventricle of the heart (almost uniformly present), and even before this occurs, increased power of the heart's action, resulting from the causes which lead up to hypertrophy. Second, anatomical changes in the kidney, which obstruct the outflow from the glomerular tuft of vessels. In order more clearly to appreciate the latter, let us first glance at the vascular arrangement in the cortex, and see how it is effected by the advancing pathological changes of granular degeneration of the kidney. The interlobular arteries, as they pass upwards towards the cortex, give off directly on either side lateral branches, the afferent arterioles, each one of which on entering a Malpighian capsule, breaks up into the capillaries composing the glomerular tuft. Now the afferent vessel of the Malpighian body, after uniting with the vessels of the tuft, pass out of Bowman's capsule, and immediately *again* sub-divide into a network of capillary blood vessels, which entwine in all directions, the convoluted tubules. This network anastomoses freely also with the capillaries of the medullary rays, forming one common net-work of the whole cortex. Now, we have before noted that the first observable pathological change in the cirrhotic kidney, is a proliferation of new connective tissue cells between and among the intertubular capillary plexus. New connective tissue cells, wherever formed, as they become old and organized, *contract,* and it thus happens, that as the disease goes on, many of the intertubular capillaries forming the plexus, become obliterated at points, while in others, they are in all stages of contraction, being choked off by the new tissue formation. It is easy to comprehend, therefore, that the outward current of blood from the glomerule, is very seriously retarded, as it is more readily assailable from its large number of branches than that of the single larger afferent arteriole, coming off as it does directly from the trunk of the interlobular artery. We thus perceive, that the blood is dammed back upon the glomerular tuft, which raises the tension especially high within these vessels. That this is no mere theory, has been proven by Dr. Dickinson, who found, upon experiment, that the granular kidney permitted much less water to pass through its vessels under pressure, than the normal organ.

It is likely that still another factor operates in the case of renal cirrhosis, to increase the intra-vascular pressure, within the glomerule, namely, the thickened capsule of Bowman.

Thus we have in the advanced stage of renal cirrhosis, both the increased force of an extra powerful contraction of the heart, on the one side, of the glomerule, and counter to this, an impeded current on the other, exerting a backward force, owing to obstructed capillaries, and these two forces meet in the glomerular tuft of vessels, and thus we have all the conditions requisite for producing transudation of albumen as well as the secretion of a copious amount of urine, of low specific gravity, all of which are the accompaniments of advanced granular kidneys, *but not till the disease is advanced.*

Having shown that albuminuria can never be relied upon as an early symptom of chronic Bright's disease, and given at length the physiological and pathological reasons therefor, as well as referring to a large collection of clinical and autopsical proofs thereof, and moreover having traced it to its philosophical and pathological cause, when it does occur; it now remains to ascertain, what are the symptoms which, taken individually and collectively, may be relied upon to establish an earlier diagnosis of the disease.

So large a proportion of the profession has become wedded to the old teachings, that albuminuria forms the hinge upon which the door to renal disease has ever been opened, that I apprehend it will be easier to explain facts, with philosophical reasoning, than it will be to do away with preconceived ideas, or break down old traditions. I am unwilling to believe, however, that a disease so widely influencing in its pathological results, undermining as it does so many of the vital processes of the economy, can approach so silently through years of development, without leaving behind some imprints, which if properly interpreted must reveal its footsteps.

First, then, let us glance at some of its more important etiological factors, for to know disease best is to first know its cause. It has been frequently pointed out that renal cirrhosis occurs most often in people who have lived very generously, partaking of highly seasoned and flavored meat diet; in short, they have been over indulgent in the pleasures of the table. It is the function of the kidneys to eliminate from the system nitrogenous waste, whether this be from the tissues or the food, or from both. We have here then a very direct cause of increased work thrown upon the kidneys, and the result is that extra activity of renal function is called for.

Notice next, that renal cirrhosis is most often developed after middle age. Statistics show that the largest number of cases occur between forty-one and fifty years of age, and the next largest between fifty-one and sixty. Thus, as a rule, it is a disease of middle and advancing life. Why is this? To my mind, Fothergill best answered this question, when he said: "The diseases incident to advancing age are those which arise through inability of the system to get rid of its waste products." Here then we have two powerful determining causes of disease, whose especial foci is the kidney: a demand for increased functional activity, and a diminished functional power.

Gout, rheumatism, free use of alcohol, and the absorption of lead into the system, as well as some other factors may, and doubtless do, act as accelerating or predisposing causes; but to my mind, around the two conditions, advancing age and ingestion of foods which leave behind the maximum of nitrogenous waste, clusters most of the phenomena of the disease, in its causative relations, and the more closely this is kept in mind, the more readily will be understood what I shall have to say of the symptoms which accompany it.

Heidenhain has shown conclusively by his beautiful experiments with indigo injections into the circulation, that the solid elements of the urine are excreted by the epithelium of the convoluted tubules. We have through heavily charged nitrogenous foods, a very material increase of work thrown primarily upon the epithelial cells of the convoluted tubules, and at a time when the functional power of such cells are on the wane, from advancing age. The cells, thus both over-taxed and crippled, become in time functionally impaired, and are unable to meet the demands made upon them. We have previously shown that necrosis of the cells does not result directly, but the impaired function is made known through the retention of those products in the system which it was their province to eliminate, and which, thus accumulating in the system, result in certain derangements and symptoms which we shall next consider.

### THE CUTANEOUS SYSTEM.

One of the earliest manifestations of chronic Bright's disease is pallor of the skin. It is pretty uniformly present in quite early stages of the disease. In some well nourished individuals, it is true, the disease does not make sufficient impress upon the blood to cause this loss of color in the skin till well advanced; but taking the average of cases, as we find them, it may be laid down as the rule that a suspicious pallor and unhealthy color of the skin is present, and we may often note this condition before albumen is discoverable in the urine. The peculiar pallor referred to is not the pronounced anæmic whiteness exactly which we meet with in acute Bright's disease, on the one hand, nor yet the semi-bronze-like hue, due to malignant cachexia, but it is rather an intermediate tint. Dickinson says: "It is somewhat of the *pallor luteus* somewhat anæmic appearance, though a sort of sun-burnt tinge upon the skin prevents the whiteness characteristic of the acute form, a dirty, faded hue."

Defective perspiration is also a common accompaniment of early cirrhotic disease, and this results in a dry, harsh state of the skin. This is probably due to two causes: compensatory elimination through renal incompetency, and perhaps later on to hypertrophy of the muscular walls of the small arteries of the skin, interfering with the nutrition of the latter.

Other cutaneous symptoms may arise, as erythema, or acne rosacea, but these are more likely to occur later in the disease.

### THE MUSCULAR SYSTEM.

One of the earliest noticeable accompaniments of chronic Bright's disease, is usually diminished muscular power. The patient experiences an unaccountable disinclination for muscular exertion, and is easily tired. He walks less, and rides more frequently than is his usual custom, when he finds it necessary to go about. A weary feeling is experienced in the muscles, and he perhaps goes to some watering place to seek rest from business, which latter, he imagines has over-taxed his strength, but he returns unimproved.

More or less emaciation is likely also to be present, though this latter does not always obtain, and, in some cases, it is conspicuously the opposite.

### THE DIGESTIVE SYSTEM.

It is well known that all forms of Bright's disease are accompanied more or less by dyspeptic symptoms, at some stage of their progress. In the form under consideration, however, dyspepsia has been noted as one of its very earliest symptoms. Dr. Geo. Johnson has observed a flatulent dyspepsia, with nausea and occasional irregularity of the bowels, to precede renal cirrhosis so frequently, that he even regards it as one of the causes of the disease. He thinks the kidneys become deranged in such cases, in consequence of their long-continued elimination of the products of faulty digestion. Be this as it may, the fact is notorious, that flatulent dyspepsia *precedes*, for a longer or shorter time, in a large number of these cases. In two cases which came under my own care, it was the first symptom which led both patients to seek medical advice, and for that which they both supposed to be simple disorders of the stomach. In one of these cases, no albumen could be found in the urine till over a year after the first dyspeptic discomforts appeared, and the latter continued, off and on, till albumen finally was discovered in the urine in small traces.* In the second case, albumen was found in the urine on first consultation, but it has been occasionally absent in the course of the disease since. Loss of appetite is an early and persistent characteristic of the disease. Nausea frequently accompanies the dyspepsia of renal cirrhosis, and when it does, it is most frequently complained of in the morning, before food is taken. Irregularity of the bowels completes the round of digestive disturbances which are the outgrowth of this disease. Constipation being the most frequent departure, and I have noted this early in those cases in which heretofore the patient had congratulated himself on most scrupulous regularity, throughout his preceding life.

### THE RESPIRATORY SYSTEM.

Dyspnœa is a frequent symptom of renal cirrhosis. It may

---

* Subsequent autopsy revealed granular kidneys.

exist in various forms, and it may be one of the first symptoms which attracts attention, or, it may not appear till ushering in œdema of the lungs, which closes the life of the patient. In the latter case, probably only in advanced stages of the disease. We may have dyspnœa coming on quite early in the disease, simulating asthma in many respects, especially in its more frequent prevalence at night. Some look upon it as an eliminative effort of the lungs. Others regard it as of cardiac origin, or at least due to cardiac disturbance. Bartels and Dickinson seem to regard the attacks as uremic, Rosenstein denies this, and Johnson speaks of them as nervous dyspnœa, of uremic origin. Whatever may be its cause, it simulates asthma closely, coming on suddenly, usually at night, accompanied by sibilant râles, and followed by more or less watery expectoration.

I am aware that many authors regard this dyspnœa as a symptom only accompanying the advanced stages of the disease. But that it may occur comparatively early in the disease, I am equally positive, as the following case has shown me. A case came under my observation in which the patient complained of dyspepsia, and attacks of short breathing, coming on at night. High vascular pressure, and other symptoms pointed to granular kidneys. The dyspnœa became much worse after a few days, indeed, in one of the spasms which I witnessed, I never saw more distress or anxiety depicted in the countenance of a truly asthmatic patient. Under diaphoretics and purgatives, these symptoms subsided, and the patient had no recurrence of them. Eight months afterwards he succumbed from outside causes, and the autopsy revealed granular kidneys by no means advanced, each organ weighing slightly more than five ounces. In another instance, three years since, that of a well-informed physician, who served in his professional capacity through the late war. He had noticed some declining health, but the first symptoms which aroused his attention, was an asthmatic attack at night, followed by others, which led to to the discovery of renal cirrhosis from which he subsequently died. It may be said, that there is no proof in the last case that the disease was not advanced, and while this is true, yet it was the first symptom which aroused any suspicions, and if no symptoms are observable till dyspnœa overtakes the case of a medical man, how much more likely is it to be the case with our patients?

That the mucous membranes are especially prone to irritation in the early stage of renal cirrhosis, we have already seen in the case of the digestive tract. It is equally so with the respiratory tract. Bronchial catarrh has been frequently noted by various authors as preceding or accompanying the granular kidney. The late Dr. Mahomed regarded this as one of the early results of high vascular pressure, which, in some instances led on to pneumonia, in people of advanced age, before they were overtaken by the renal disease. Pharyngeal or naso-pharyngeal catarrh, is another evidence of disturbance of the respiratory tract, which may often be observed in early cirrhosis; and, lastly, I would mention, the frequent occurrence of emphysema of the lungs in these cases.

THE VASCULAR SYSTEM.

Of all the disturbances which arise in the course of chronic Bright's disease, those of the vascular system are among the *earliest, most constant*, and give us, when properly interpreted, the most important information. If the theory which we have advanced as to the etiology of the disease, and supported thus far by pathological evidence, be correct, then the first consequence which results from the disease, outside of the kidneys themselves, must fall upon the vascular system. And it will be found on following its pathology a step further, that the deduction will be confirmed.

Dr. George Johnson pointed out* certain interesting and constant anatomical changes, which occur in the minute arteries in cases of granular degeneration of the kidneys, consisting of thickening, and hypertrophied growth of their muscular walls. He showed that both the circular and longitudinal muscular layers of the small arteries constantly participated in this hypertrophic development, but that the veins do not share in this change. Dr. Johnson regards this change in the walls of the small vessels as due to the resistance offered in the capillaries to the circulation of abnormal blood. He says: "The minute arteries, by their contractile power, under the influence of vaso-motor nerves, now regulate the blood supply in accordance with the diminished requirements of the glands. This regulating contraction continues and increases, month after month, year after year, and the physiological result of this persistent over-action of the minute arteries is that their muscular walls become hypertrophied." He refers the hypertrophy of the left ventricle of the heart (so constantly accompanying this disease) to a similar cause, for he adds: "The minute arteries throughout the body (of course under the influence of vaso-motor nerves) resist the passage of this abnormal blood, and in consequence the left ventricle beats with increased force to carry on the circulation. The result of this antagonism of forces is, that the muscular walls of the arteries, and those of the left ventricle of the heart become simultaneously hypertrophied."

Gull and Sutton, later on, claimed, 1st, "That there is a diseased state of the arterioles and capillaries in this disease, characterized by increased growth in the outer fibroid coat, or

*Medico-Chirurgical Transactions, Vol. xxxiii.

*tunica adventitia,* which they termed *arterio-capillary fibrosis.*"

2d. "That this change in the arterioles and capillaries is the primary and essential condition of the morbid state called chronic Bright's diseases with contracted kidney."

3d. "That the morbid state under discussion is allied with the conditions of old age, and its area may be said hypothetically to correspond to the *area vasculosa.*"

4th. "That it is possible that this change commonly begins in the kidney, but that there is evidence of its beginning primarily in other organs, and finally, that the contraction and atrophy of the kidneys are but a part and parcel of the general morbid change."

In the light of subsequent investigation, the only one of these propositions of Gull and Sutton which remains tenable is the existence of fibroid change in the small vessels, *not to the exclusion of,* but in addition to, the muscular hypertrophy.

That the cardio-vascular changes are dependent directly upon renal deficiency, is shown, 1st, by their constantly accompanying all forms, even scarlatinal, of that deficiency, if only it continues long enough, or the patient survives a sufficient length of time; and this becomes another proof, that in cirrhotic kidney the functional incapacity of the organ may long exist before it is made known by very active general symptoms.

2d. That the occasional existence of the disease in children, in which cases all the typical cardio-vascular changes accompany it, completely disproves the second, third and fourth propositions of Gull and Sutton, and moreover, furnishes the very strongest evidence that the cardio-vascular changes are subject to the renal disease, as their primary, unvarying, and constant cause.

Professor Hamilton, of Aberdeen,* has formulated the laws,

* "On the Circulation of the Blood Corpuscles Considered from a Physical Basis."—Journal of Physiology, Vol. v., No. 2.

which govern the circulation of the blood corpuscles in the vessels, in the most lucid manner, and demonstrated their correctness by means of a series of interesting illustrations. These consist in passing a current of water, containing bodies of varying sizes, shapes, and specific gravity, through glass tubes, which thus imitate the systemic circulation. He first calls attention to the fact, that the blood current consists of an axial and peripheral stream, and that "the colored corpuscles float exclusively in the axial stream, while a great many, not all, of the leucocytes run in the peripheral."

a Vessel wall.

b White corpuscles in peripheral stream.

c Red corpuscles in axial current.

He demonstrates clearly the following among other laws by practical experimentation:

1st. "That if a sphere is specifically lighter than the liquid

1 Web of frog's foot

2 White blood corpuscles.

After Hamilton.

in which it is suspended, it will sooner or later come to occupy the upper strata, and will rotate."

Now this is precisely what the colorless corpuscles do in normal conditions of the blood, as may be demonstrated by observing the circulation in the frog's foot, viewed horizontally by means of the microscope. The white corpuscles will be seen to roll along the upper surface of the vessels, because they are lighter than the blood plasma in which they move.

2d. "That a disc or sphere of the same specific gravity as the liquid in which it is immersed, moves in the axial stream, and *does not rotate.*"

This explains why the red corpuscles, as they are of the same specific gravity as that of the blood serum, occupy the axial stream of the vessels, and do not rotate as do the colorless ones, but *glide.*

3d. "Spheres, or discs, whose specific gravity is greater than the fluid in which they circulate, move in the lowest strata of the tubes, and *roll*, or rotate."

Thus it will be noted, that bodies which depart from the specific gravity of the fluid in which they circulate, occupy the peripheral stream and rotate as they move; while those which do not depart materially from the specific gravity of the circulating fluid, move in the axial stream, and do not rotate, but glide.

Other laws are formulated and illustrated in the same paper bearing on the size, weight, and rapidity of motion, as the bodies occupy the axial, or peripheral stream, and the fact is noted, that the colorless corpuscles evince a tendency to stagnate, or clog in places, especially at the curves of the tubes. I have gone over these experiments with Professor Hamilton in his laboratory, and I know that the laws which he formulates do not rest on any mere theoretical basis, but are amply borne out by his interesting and instructive demonstrations. Professor Hamilton thus sums up this matter: "The cause of a colorless corpuscle blocking the tube undoubtedly is, that its light specific gravity tends to press it upwards against the wall of the vessel, to which it may become temporarily adherent. From this we may learn an important lesson, namely, that if the colored corpuscles were not so balanced as to closely approach the specific gravity of the plasma, the circulation would become a physical impossibility; for if they were specifically lighter or heavier than the plasma to a marked degree, there would be a constant tendency for them to obstruct the capillaries and to hinder the onward flow. The essence of the blood circulation is that the large majority of the corpuscles never touch the wall of the vessel, but glide in the central stream. Were the colored corpuscles to rub against the wall of the vessel, the friction would be so enormous over the whole capillary system that the heart, as at present constituted, would be wholly inadequate to drive the blood onwards. Indeed, my own impression is that if the blood corpuscles all differed materially from the specific gravity of the plasma, the circulation could not be carried on even with a much more powerful heart."

Now, what is the actual condition of the blood in cases of granular degeneration of the kidneys? From the various sources from which I have been able to get statistics, I have estimated, roughly, that the blood is about ten per cent. more hydremic in cases of renal cirrhosis, than in the normal condition. It must not be forgotten, that other circumstances influence the deviation from the normal, in this disease, but all observers agree that it is somewhat lighter than in health. My own calculation from the few tables I have been able to find, places the average specific gravity of the blood serum in renal cirrhosis at 1025.5. These estimates, in the light of Professor Hamilton's researches, teach us, to say the least, a very probable explanation of that interesting and important series of cardio-vascular changes, which so uniformly accompany the granular kidney.

Not only may the loss of balance between the plasma and corpuscles call for more muscular development in the fibres of the heart, resulting in hypertrophy of the ventricle, but if also, as Professor Hamilton points out, even a part of the red corpuscles of the blood are forced into the peripheral stream, they may by their friction against the vessel walls be the source of an irritation, which gives rise to the degenerative changes therein.

It has occurred to me that Professor Hamilton's laws may explain a phenomenon which is often present in renal cirrhosis, and which, heretofore, I have never been able to explain satisfactorily to myself. I refer to the singular circumstance that in such cases the patients often pass much larger quantities of urine at night than in the day time. It now seems plain enough to me, that if the blood plasma is reduced in density, when the patient assumes the recumbent position more of the red corpuscles gravitate into the peripheral stream, and the extra friction induced thereby, calls for more force of the heart, and thus the intra-vascular pressure becomes high, and this latter we know always increases the quantity of urine.

We may not as yet be able to comprehend all the causative relations of these vascular changes, but we are very certain of one fact, namely, that increased intra-vascular pressure and high tension of the vessel walls are the most constant of all the early symptoms of this disease. As indications of these conditions, I would call attention to the character of the pulse, which is hard, unyielding, and rolls under the finger like a cord. The pulse is notably prominent under the finger in chronic Bright's disease. But high pressure of the vessels is estimated much more accurately by means of the sphygmograph, and hence this instrument has proven of the greatest value in pointing out the true condition of the circulatory apparatus in chronic Bright's disease.

Fig. 5.

Fig. 5 is the pulse tracing of a healthy man, under three ounce pressure, and may be fairly said to represent the usual pulse tracing by the sphygmograph, in perfectly healthy conditions of the heart and vessels.

Fig. 6.

Dr. Mahomed estimated high tension as follows: First. "A line must be drawn from the apex of the up stroke, 1, Fig. 6, to the bottom of the aortic notch, 2. No part of the tracing should rise above this line; if it does, then the pulse is one of high pressure." Second. "The height of this notch is another good gauge of pressure, the higher it is from the respiratory line AA, the higher is the pressure; the nearer it approaches the respiratory line AA, the lower is the pressure." The length of the systole of the heart, 5 to 6, Fig. 6, compared with that of the diastole, 6 to 7, is also considered a gauge of high pressure. In normal tracing the length of the systole, as marked on the respiratory line, AA, Fig. 6, should be about half as long as that of the diastole. In conditions of high pressure, the systole is lengthened, in some cases exceeding the length of the diastole.

I think it is unfortunate that the terms high tension and high pressure have been so much confounded with each other by nearly all authors.

I consider a pulse of high tension one in which the vascular walls are rigid and inelastic from organic change. It may or may not include high vascular pressure. The term high pressure I define as increased intra-vascular force, coming from increased power of the heart, or resistance in the capillaries.

Dr. Byrom Bramwell* says: "I am in the habit of considering a pulse of *high tension* as synonymous with a *strong pulse* and *vice versa*, a pulse of low *tension*, with a weak pulse."

Now, the above does not by any means follow, as I will illustrate. Fig. 7 shows the pulse-tracing of a patient of mine, taken March 8th, whose case I had diagnosticated eight months previously as cirrhosis of the kidneys.

Fig. 7. 4 oz. pressure.

It will be observed that this tracing shows in a marked manner the characteristics of high tension. The tidal wave is sustained above an imaginary line from apex of percussion stroke to

* "Students' Guide to the Examination of the Pulse and use of the Sphygmograph, 1883."

the bottom of the aortic notch. About two days after this tracing was taken, March 11, the patient was suddenly seized with gastric hemorrhage, losing a large amount of blood (over a pint) the first twenty-four hours. The hemorrhage continued interruptedly, sometimes ceasing for a day or so, till March 26, when he died, at 3 A. M., from direct loss of blood.

The autopsy revealed granular kidneys, though not advanced to contraction, atheromatous arteries everywhere, including that of the aorta. No appreciable hypertrophy of heart. Behind pylorus of stomach a scirrhous mass, which latter doubtless was the cause of the hemorrhage.

Now, no one will pretend to claim that high intra-vascular pressure could be present after the first great loss of blood, much less as the hemorrhage progressed day after day, till the patient died from anæmic exhaustion, yet it will be noted throughout that the tracing maintained the usual characters of high tension, even when the characteristics of high pressure were abolished and the pulse became somewhat dicrotic. Nor

Fig. 8.

Fig. 9.

Fig. 10.

Fig. 11.

were the indications of tension lost till the pulse became even hypodicrotic, as shown by the tracing of Fig. 11, taken a few hours before death.

This case has shown me that we may have tension or rigidity of the vessel walls, with very ordinary, or indeed low intravascular pressure. High tension, then, is due to rigidity of the vessel walls, from atheroma, or allied conditions, and it is presumptive evidence of chronic Bright's disease. I may state that I have never failed to find the characters of high tension in the tracing of a patient's pulse in renal cirrhosis, no matter how early the form. It is not positive evidence of chronic Bright's disease, because we have atheroma aside from Bright's disease sometimes, and if we can separate the causes, it will give us pretty positive evidence.

High pressure includes high tension, and is due to powerful and prolonged contraction of left ventricle of the heart, frequently with hypertrophy of the latter, and as such is almost pathognomonic of chronic Bright's disease.

Fig. 12.

Fig. 12 shows the characters of the tracing of high pressure. The prolonged systole, 1 to 2, and 3 to 4, as compared with the diastole 2, 3, and 4, 5. It shows also the high and sustained tidal wave c. c., and the aortic notch e. e., high and well away from the respiratory or base line A. A. For comparison with characters of tracing in simple high tension, see fig. 6.

The late Dr. F. A. Mahomed collected the records of sixty-one patients, treated in Guy's Hospital during the years 1879 and 1880, for chronic Bright's disease. They possessed the following characters: "*They all had the signs of high arterial pressure.* They all had very considerable hypertrophy of the heart. *In all cases the urine was free from albumen at some time while under observation.* In eleven cases albumen was present on one or two rare occasions during a long period of observation. In three cases, though absent during long periods of observation, it occurred just previous to death; in three other typical cases of Bright's disease, the patients were admitted with albuminuria, which disappeared under treatment, and they left without it. Three cases had urine very variable in its character, sometimes albuminous, sometimes not. In the remaining forty-one cases, *albumen was never discovered in the urine.*" It may be added that in *all* these cases daily observations were made. The quantity, specific gravity, solids, and albumen present in the urine, were appended.

It is the rule, then, that high arterial pressure invariably accompanies chronic Bright's disease, and that it precedes the albuminuria.

Dr. Mahomed found high pressure to precede the disease so invariably, and in some cases so long, that he was led to believe that it was the *cause* of chronic Bright's disease, at least in some cases. I cannot believe that this view is in keeping with the clinical history or pathology of the disease, and moreover, if it were correct, bleeding and purgatives should arrest the disease.

Epistaxis of an obstinate character is another symptom of chronic Bright's disease to be ranged under those pertaining to the vascular system. It also is due to degenerated vessels, and high arterial pressure, which accounts for its obstinacy. In some of my cases, inquiry into their history has revealed the occurrence of an obstinate attack of bleeding from the nose very early, and I attach considerable importance to such occurrences. Epistaxis, due to renal causes, may sometimes occur, as far back as two or three years, or more, before any appreciable symptoms of the disease are manifest. Vertigo is also one of the early symptoms which is well to note the import of. It may be so pronounced that the patient may fall in walking, as was the case with one of my patients more than once before its cause was made out. Tortuous arteries, as the radial and temporal, quite uniformly accompany renal cirrhosis and are manifest quite early. I have noted small aneurisms of superficial arteries, particularly in the wrist and neck, as sometimes present. I have not seen this noted in the literature of this disease, but it has occurred in my cases often enough to call my attention to it as a sign. The *superficialis volæ*, in the wrist, and small branches of the external carotid are the most usual seat of these small aneurismal sacs, according to my observations.

THE NERVOUS SYSTEM.

Valuable as are the symptoms referable to the vascular system in an early diagnostic point of view, those of the nervous system are scarcely less so. Among the most important of these are retinal changes and visual disturbances.

Retinal changes are often the first symptom leading to discovery of the disease. Bartels says: "Very often some disorder of vision, provoked by the specific structural change that has taken place in the retina, forms the first event that attracts the patients own attention. No small number of my patients first went, on account of their eyes, to my colleague Voelckers, and were first induced by him to place themselves under medical treatment, which they were not aware they needed." The frequency of these retinal changes in the course of renal cirrhosis are such as to constitute them important occurrences to note. Eale's* statistics, founded on observations of the *fundus* in one hundred cases of granular kidneys, show the presence of retinal change in twenty-eight, or one in three and a half.

Gowers says: "The retinal disease presents certain elements which are variously combined in different cases. These are (1), diffuse slight opacity and swelling of the retina, due to œdema of its substance; (2) white spots and patches of various sizes and distribution (fan shaped), due for the most part to degenerate processes; (3) hemorrhages; (4) inflammation of the intra-ocular end of the optic nerve; (5) the subsidence of inflammatory changes may be attended with signs of atrophy of the retina and nerve."

The degenerative is the most frequent form, beginning in small white spots, which spread out in fan shape near the *macula lutea.* This leads to impairment, *not complete loss,* of vision, and comes on slowly, and is rarely, if ever, completely recovered from, though improvement is common. The hemorrhagic form comes on very suddenly, as a rule, and often results in total blindness. It may be recovered from completely, or induce inflammatory changes.

Gowers has made the important observation† that in many cases of chronic Bright's disease, there is to be seen a notable diminution in the size of the retinal arteries, *independently of the existence of any special retinal disease.* He says: "The veins are in such cases not larger than the normal, but the arteries are *not more than one-half, or even one-third, the diameter of the veins,* instead of being two-thirds, or three-quarters

*Birmingham Med. Review, January, 1880.

†British Medical Journal, Dec. 9th, 1876.

the diameter. The comparison must be made between arteries and veins which run side by side, and correspond in distribution." I regard the above discovery as only second in importance to the indications afforded by the sphygmograph in cases of chronic Bright's disease.

Other symptoms of nervous disorder are more or less frequently present in early cirrhosis. Neuralgia is perhaps the most common. It is most often occipital, but may be temporal or vertical. In patients, without previous history of neuralgia or assignable cause, it is important to note such symptoms. Insomnia is frequent, and marked in a large number of cases.

THE URINARY SYSTEM.

Often the first symptom that the patients notice is that they get up at night to urinate. This, as I have previously noted, is because more urine is secreted at night than during the daytime. Usually before the urine contains albumen, if observed it will be found of high specific gravity, say from 1.020 to 1.035. And here I would insist, that the specific gravity of any single specimen of urine, unless it be a part of the whole twenty-four hours' quantity, is entirely valueless from a diagnostic point of view. The urine must be saved during the whole twenty-four hours, and the specific gravity compared with both the normal gravity and quantity. If found constantly much over 1.020, the fact becomes important. The urine throws down, as a rule, numerous envelope-shaped crystals of calcium oxalate, and also uric acid crystals; in fact, the urine is often loaded with urates in these cases. Lastly, the urine may give the blue color reaction with peroxide of hydrogen solution and tincture of guaicum, showing the presence of blood crystalloids. This latter is important when present, but it is not so constant as the others. Such, in the main, are the symptoms most likely to be met with in the pre-albuminuric stage of chronic Bright's disease. Our diagnosis is rather to be sought through their collective than individual study, for it is only upon a thorough comprehension of all the phenomena surrounding the morbific changes of this disease, that it becomes possible to penetrate its varied symptomatology during its incipiency.

All life, and growth is influenced, not only by the soil or pabulum which sustains and nourishes it, but also, by the inherent qualities of the seed or germ from which it sprang; so with disease and decay, not only are they maintained by the lesion of the individual organ, but also influenced by inherent inherited qualities; they follow no stereotyped laws of symptomatology in all cases, but the first manifestations become apparent in this location in one case, and in the next location in another, as one or the other part of the organism is inherently weak, or has been unduly taxed. Chronic Bright's disease is no exception. In one class of patients, the results of impaired renal function will first fall upon the circulatory system, while in another, it may be on the nervous, the respiratory, the digestive, or the urinary system itself. Our diagnostic conclusions should hence be drawn, not alone from the local symptoms, as perhaps has been too much the custom, for if we wait for marked evidences of impairment here, the disease will often escape detection till the anatomical changes become so far advanced that the disease will be practically beyond the reach of medication. The textural changes, from their very nature, are such, that when once thoroughly engrafted upon the organ, their removal is not within the compass of the present state of medical science, and hence our only hope of arresting this disease, must rest on its discovery during the functional stage. If, fortunately, I shall have contributed in any way to the elucidation of the latter question, the object of this paper will have been accomplished.

163 State Street.

## Figures and Tables

**Fig. 1. f1:**
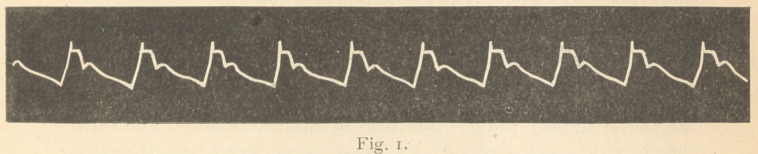


**Figure f2:**
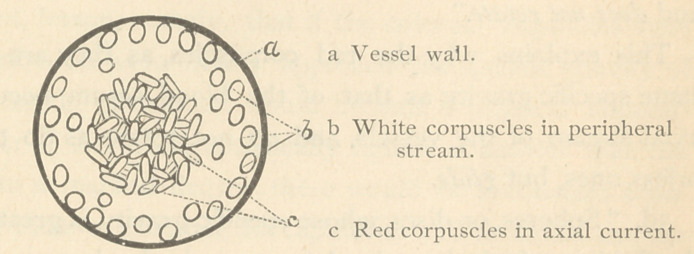


**Figure f3:**
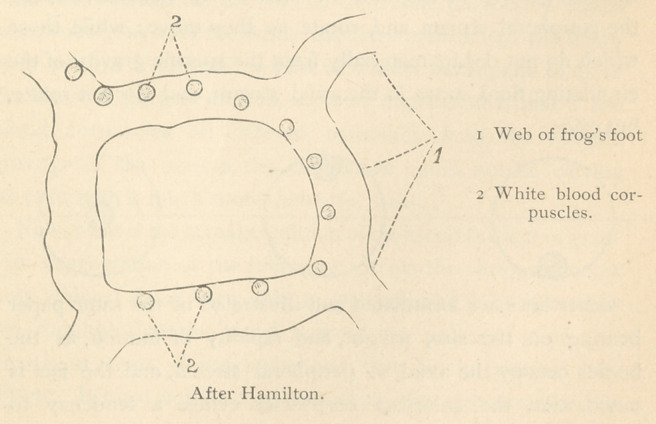


**Figure f4:**
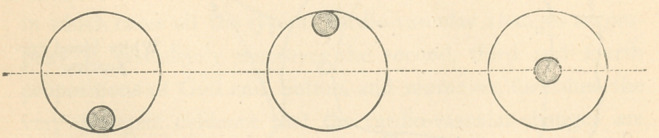


**Fig. 5. f5:**
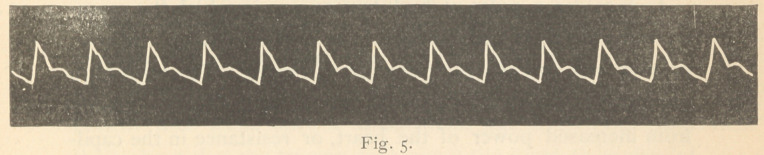


**Fig. 6. f6:**
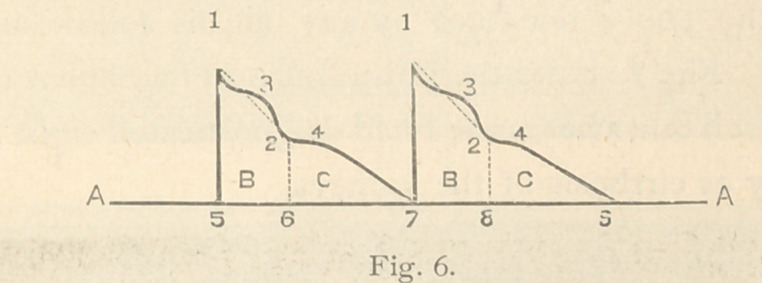


**Fig. 7. f7:**
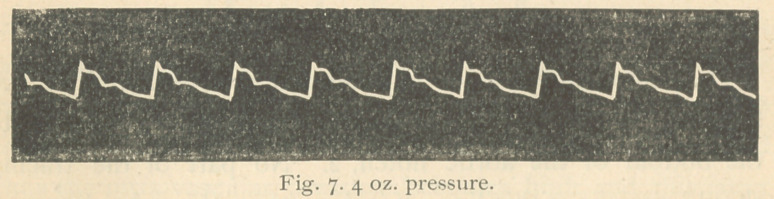


**Fig. 8. f8:**
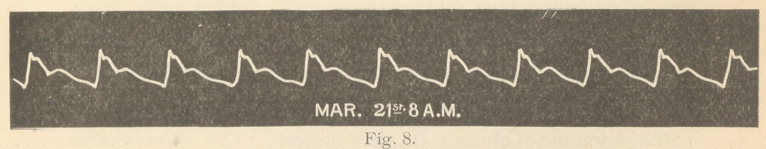


**Fig. 9. f9:**
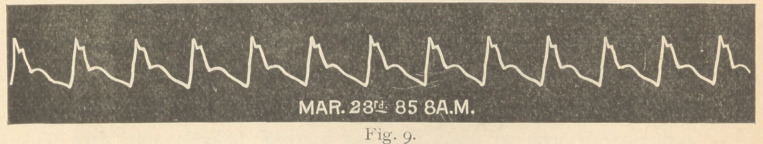


**Fig. 10. f10:**
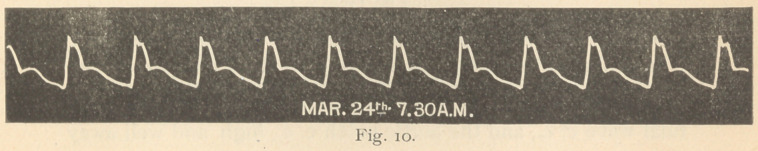


**Fig. 11. f11:**
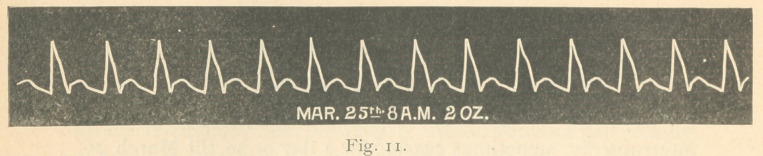


**Fig. 12. f12:**